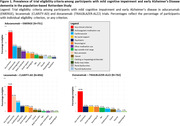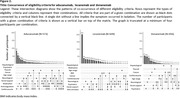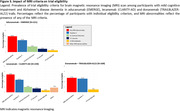# External validity of trials on amyloid‐lowering therapy against Alzheimer’s disease: the population‐based Rotterdam Study

**DOI:** 10.1002/alz.088706

**Published:** 2025-01-09

**Authors:** Jacqueline Josephine Claus, Ilse vom Hofe, Annekee van Ijzinga Veenstra, Silvan Licher, Harro Seelaar, Frank Jan De Jong, Julia Neitzel, Meike W. Vernooij, Arfan Ikram, Frank J. Wolters

**Affiliations:** ^1^ Erasmus Medical Center, Rotterdam, Zuid‐Holland Netherlands; ^2^ Erasmus University Medical Center, Rotterdam Netherlands; ^3^ Erasmus Medical Center, Rotterdam, Zuid Holland Netherlands; ^4^ Erasmus MC University Medical Center, Rotterdam, Zuid Holland Netherlands; ^5^ Department of Neurology, Erasmus Medical Center, Rotterdam Netherlands; ^6^ Department of Neurology and Alzheimer Center, Erasmus Medical Center, Rotterdam, Zuid Holland Netherlands; ^7^ Department of Neurology & Alzheimer Center Erasmus MC, Erasmus Medical Center, Rotterdam Netherlands; ^8^ Harvard T.H. Chan School of Public Health, Boston, MA USA; ^9^ Department of Radiology and Nuclear Medicine, Erasmus University Medical Center, Rotterdam Netherlands; ^10^ Erasmus University Medical Center, Rotterdam, Zuid Holland Netherlands; ^11^ Erasmus University Medical Center, Rotterdam, Zuid‐Holland Netherlands; ^12^ Department of Epidemiology, Erasmus MC, Rotterdam Netherlands

## Abstract

**Background:**

Treatment with monoclonal antibodies against amyloid‐β slowed cognitive decline in recent randomized clinical trials in patients with mild cognitive impairment (MCI) and early dementia due to Alzheimer’s disease (AD). However, trial eligibility criteria may affect generalizability to clinical practice.

**Methods:**

We extracted eligibility criteria for trials of aducanumab, lecanemab and donanemab from published reports, and applied these to participants with MCI and early clinical AD dementia from the population‐based Rotterdam Study. Participants underwent routine, standardized assessment from 2002‐2013, including questionnaires, blood sampling, brain MRI, cognitive testing, cardiovascular assessment, and –in a subset– amyloid‐PET. We determined trial eligibility for all participants, and assessed progression to dementia within 5 years among participants with MCI.

**Results:**

Of 968 participants (mean age: 75 years, 56% women), 779 had MCI and 189 early clinical AD dementia. Around 40% of participants were ineligible because of amyloid negativity, similar across trials. Overall, at least one clinical exclusion criterion was present in 76.3% of participants for aducanumab, 75.8% for lecanemab, and 59.8% for donanemab. Criteria that most often led to exclusion were cardiovascular disease (35.2%), use of anticoagulants (31.2%), psychotropic or immunological medication (20.4%), psychiatric disease (15.9%), and lack of social support (15.6%). One‐third of participants were ineligible based on their brain MRI alone, which was similar across trials and due predominantly to various manifestations of cerebral small‐vessel disease. Combining amyloid, clinical, and imaging criteria, eligibility ranged from 8% for aducanumab and lecanemab to 15% for donanemab. Risk of progression to dementia tended to be higher for ineligible than for eligible participants for lecanemab (hazard ratio [95%CI]: 1.64 [0.92‐2.91]), but not aducanumab (HR: 1.17 [0.65‐2.12]) or donanemab (HR: 1.03 [0.67‐1.59]).

**Conclusions:**

Less than 15% of community‐dwelling individuals with MCI and early AD dementia would have been eligible for participation in recent RCTs of monoclonal antibodies against amyloid‐β, due in large part to comorbid vascular pathology. These findings underline that evidence for drug efficacy and safety is lacking for the vast majority of patients with AD in routine clinical practice.